# Extra Supplement.—The Nursing Mirror

**Published:** 1888-12-01

**Authors:** 


### The Nursing Mirror.

All Contributions for this Supplement should be addressed "The Nursing Mirror," care of The Editor, 38, Old Jewry, E.C.

### En Passant.

CHARING CROSS.—In our notice of Miss Gordon's appointment as matron to Charing Cross Hospital, some figures were given which unluckily applied to Guy's Hospital. Charing Cross has only 115 beds occupied on an average; and 33 nurses and nine sisters make up the nursing staff. We are obliged to those correspondents who pointed out our error, which was such an obvious one we can only hope it misled no one.

THE PRICE OF ATTENTION.—At the late meeting of The Hospitals Association at Barts, when a great number of nurses were present, Mr. Lushington, the treasurer of Guy's, said a few kind words about the improvement in nursing which is daily becoming more pronounced. He said his hearers would remember how Dickens described that when a patient complained that the nurse had rubbed the soap into his mouth, the nurse replied, "How can you expect all your features to be picked out, and all manner of fine work done for half a-crown a-day?" And the old story was received with laughter from the audience.

LADY NURSES.—A book called "Chats at St. Ampelio," which has been lately published, contains some remarks on nurses worthy of attention; especially as their author is the well-known Dr. J. A. Goodchild. In reply to a question as to whether doctors like lady nurses the answer is made: "Lady nurses, yes; lady interlopers, no." And the difference between these two is then discussed. The pride of craft amongst modern nurses which leads them to question the doctor's treatment and snub the patients' friends is first pointed out; then the second great mistake as to the light in which to regard nursing is dealt with—the mistake which causes a woman to regard the performance of her duties as a priestly sacrifice, and her patient as so much material for the discipline of her soul—just as fasting and scourging are regarded. Both these types of women are well known in nursing ranks, the craftswoman and the religious bigot, and care should be taken not to fall into the mistake of either.

BRITISH NURSES' ASSOCIATION.—The following official notice is being issued with the complimentary cards of invitation:—

"A conversazione will be held on Friday, December 7th, at 9 p.m., at the Grosvenor Gallery. The President, Her Royal Highness Princess Christian, has announced her intention to be present. The gathering will be most picturesque, as several hundred nurses will probably be present, wearing the uniforms of their different hospitals. The Pastel Exhibition will be on view, and also an Exhibition of Nursing Appliances. A short ballad concert and other entertainments will be given. To afford a few—of the many—interested in nurses and nursing an opportunity of being present on such an interesting occasion, a limited number of cards of invitation (price half-a-guinea each) can be obtained by early application to the hon. secretaries, 20, Upper Wimpole Street, W."

There is nothing so popular with certain people as a conversazione, and the last one given by The Hospitals Association, though organised at short notice, was too crowded to be pleasant. With months of organisation and the Princess Christian's presence (if there is really life in the B.N.A.) there should not be an inch of unoccupied space at the Grosvenor. In any case it is very good of the Princess to display so much interest. Indeed she is deservedly famous for the energy which she throws into everything she takes up. This is happily the first time, however, as we hope it will be the last, that "several hundred nurses in uniform" have been publicly exhibited for the inspection of those who care to pay half-a-guinea for viewing this "picturesque gathering."

NEWS FROM ABROAD.—The first Danish lady missionary has lately gone to India: she is a Deaconess and a trained nurse.—Mdlle. Rault has received the French Prize of Virtue of £80. She has long been engaged in charitable work in Paris, including nursing, and is now busy teaching gutter children to make umbrellas.—Senorita Rerez has a large practice in Valparaiso; she took her M.D. degree at Santiago.—A National Woman Physicians' Association has been started in America, with the object of mutual aid for all medical women.

THE WOMEN'S JUBILEE OFFERING.—Until November 30th the pearl and diamond necklace presented to the Queen by her Women Subjects, will be on view to subscribers, at 130, Regent Street. The Jubilee Offering has now been nearly brought to a close; the colossal statue of the Prince Consort is nearly finished, and a quarter of a million copies of a letter of thanks from the Queen have been distributed to contributors. As to the £70,000 handed over to help forward the work of nursing, the scheme is being carefully worked out, and results will soon be seen. The word "Jubilee" is now no longer a bugbear, but is fading away from our daily conversation, yet, to charitable institutions it must leave a very pleasant memory. It may be long ere England see another burst of philanthropy such as that with which she celebrated the Jubilee of Queen Victoria.

TRAINED OR NOT?—Very naturally, Dr. Bostock's remarks about the Chichester Infirmary nurses are causing quite a commotion in that town; and the supporters of the infirmary are dividing themselves into two parties on the subject. The following extract from the report just issued will largely explain matters: "The Committee consider that this is in a great measure due to Miss Bodger, and that they ought not to allow an opportunity to pass without giving expression to their appreciation of her excellent management. Moreover, it appears that by her energy and perseverance and by her business capacity she has earned for the infirmary in the last three years a profit of about £400." The matron has evidently been doing her best for the infirmary; it has stood first in all her plans, and perhaps a little more thought for nurses and private patients might have saved the present unpleasantness. It is always a pity when a report has to descend to quoting such testimonials as the following: Nurse Baxter—"Much pleased with her services: she has been attentive, kind, and efficient. Conduct, efficiency, and attention entirely satisfactory. A very efficient helper, and very satisfactory in her conduct, and obliging and kind in her manner to all the household." Nurse Beal—"Gave great satisfaction. Most kind and attentive to her patients, and did everything in her power for them. Very much liked." As a matter of fact, the nurses do seem to have been very much liked by their private patients, but a very pleasant attendant is not synonymous with a hospital-trained nurse, and the infirmary advertises to supply the last, which is the greater, and should, therefore, contain the first, which is the less. "A Lover of Justice," who writes to the *Sussex Daily News*, tells some tales about the inefficiency of the nurses, which are truly extraordinary. It can only be hoped that the Infirmary Committee will now see the folly of trying to increase their funds by supplying private patients with inferior nurses. It is not businesslike, and is a great financial mistake. No institution should send out any nurse who cannot show a thoroughly good certificate of training. With an average of 42 occupied beds, the infirmary ought to be able to train its own nurses in three years.

### Original Articles.

### ONE WAY OF NURSING.—Part II.

By an Australian Nurse.

We were anxious to have a nurses' home, properly furnished and provided, in a central part of the city; and to make it a condition that the nurses should obey to the letter a code of rules drawn up for them, and wear a convenient uniform. But we had, so to speak, no show at all. The hard-headed, heavily-cashed ones had all the innings. There was much talking done at this meeting—which goes without saying, and to record which would not tend to edification—but it ended in our collecting a large sum of money, with ample promises of more to come, and in our launching on the world, with much travail, this big committee; with a hon. secretary of committee (a Fellow of his college), an hon. secretary of sub-committee, half-a-dozen other office-bearers, and sundry books of rules and regulations—and for what?

For the purpose of "running" two good creatures of the lay-help gangs order, who are of excellent character, and have received some training—where or of what length it boots not to say. These each receive a salary of £100 per annum, and are under the superintendence of a sub-committee of ladies and gentlemen, whose practical nursing knowledge is for the most part coeval with their term of office; and whose duties are, to accompany on the rounds at certain or uncertain intervals, and to report on the work of a woman—presumably an adept. The nurses were to live in lodgings within a reasonable distance of their work.

The idea of a uniform unfortunately grating on the feelings of the nurses, it was not pressed on them. In lieu of it they wore, as a badge of their order, a small red cross, sewn on one sleeve, and generally hid under the cloak. (This was changed subsequently, the wearing of a uniform being made compulsory.)

It was also decreed that relief should be given—and it was and continues to be, largely and without discretion—by nurses and ladies. Into the bargain, during their slack times the nurses act as fetchers and carriers of medicines and lotions, which, considering the well-known and universal prolificality of the poor, and the crowds of gaping idlers round every corner (with every one of them a good-natured spot in him, if only one will take the trouble to touch it), seems a direct waste of energy.

Then the nurses are allowed to make frequent calls at houses, "to look people up," or to "speak a kind word to them," or to leave beef-tea or other comforts, without doing a stroke of nursing in these places; so that frequently it must be very confusing to the visitor to discover what the primary occupation of the visitor really is.

This system of relief-giving has cut us off completely from carrying our work into the homes of the decent artisans, who, looking on the nurses as mere purveyors of slops to paupers, will have none of them. Moreover, they desire neither the presence nor the money of finely-dressed ladies. As for the doctors who, under other conditions would, I am convinced, give us ample work; they, with the exception of a very limited number, know nothing whatever about our nurses—who naturally cannot visit the different medical men, to explain their work and to ask for cases, and obtain the hearing that would be at once accorded to educated women. When our nurses do call, they somehow manage, as a rule, to obtain only the sort of patients that require nourishment, and that any ordinary handy woman could look after, certainly not those that require skilled nursing; and so we have to seek our work in the very lowest quarters, often in huts, where, if real sickness occurs, the only possible thing to do is to pack up the case and convey it with all speed to the nearest hospital. Therefore our cases are mostly chronic ones, bronchitis, simple "rheumatiz," "asthmatics," debility (a very convenient term), and bad legs innumerable, and my experience when I sat on the sub-committee and took my portion of superintending with the rest, was that the nurses' chief time went in pursuing these legs. They seemed ubiquitous; they were rarely at home; exercise seemed to suit them, for so sure as we were directed round one corner the leg had invariably slipped round the next, and we generally caught it at last only by making the circuit of a good half-dozen streets. I can honestly bear testimony that if the direct nursing duties of our nurses are light, it is taken out of them thoroughly in walking and carrying. But, as far as I can judge, the district nursing of Melbourne, as it stands at present, would be as efficiently done by a half-score of girls for the love of God, as at the expense of three or four hundred a-year: not to mention the "honest sweat" of over a dozen Australian matrons, and the men, lay and clerical, that make up the number of the committee. And yet the pity of it is that the need for trained and organised nursing for our poor is a crying and a most urgent one, and is likely to remain so until experience has opened many a pair of eyes, and taught many a hard lesson.

### Rules for Nurses.

The following rules of the Nursing Institute newly started at Taunton will interest many of our readers:—

7—The establishment shall consist of i. Lady superintendent of nurses; ii. Ward nurses; iii. Certificated nurses; iv. Nurses; v. Pupil nurses.

8—The lady superintendent of nurses shall be elected by the general and medical committees according to rule No. 57 of the hospital rules.

9—Candidates desirous of receiving training in this institute should apply to the lady superintendent of nurses. A statement as to age, health, character, etc., according to the form which will be supplied, must be sent in, and, as vacancies occur, candidates will be chosen by the institute committee. They shall serve one month on probation, and will then, if found suitable, be engaged. The age considered desirable for entering is from twenty-one to thirty.

10—Candidates who are engaged shall be called for the first year "pupil nurses," for the next two years "nurses," afterwards "certificated nurses," if passed as qualified by the medical committee.

11—The names of nurses should be entered in a register, in which a record will be kept of their conduct and qualifications.

12—After serving one month on trial the candidates accepted, and who sign the agreement for three years, shall receive £10 for the first year, £14 for the second year, £18 for the third year. Those who at the expiration of this term take their certificate, and remain in the service of the institute, will receive £20 for the next year, with an advance of £1 a-year until the salary reaches £24. They will be supplied with board, lodging, uniform, and a reasonable amount of washing.

It is also proposed to employ five ward nurses at a salary of £25 a-year each.

13—Nurses will be employed as required either in hospital, private, or district nursing.

14—All nurses will be expected to attend some place of public worship on Sundays.

15—Nurses will be provided with indoor and outdoor uniforms, which must be worn on all occasions except during their annnal holiday. The uniforms are the property of the institute.

16—Nurses are not permitted to receive any private remuneration in money or clothing; a book or other article of small value as a token of remembrance of a patient may however be accepted without infringing this rule.

17.—Nurses are to keep the apartment alloted to their use neat, clean, and in proper order. They are to sweep and dust, and also to be generally useful in the wards as well as to assist in the needlework there.

18—No nurse is to return from her employment without permission from the lady superintendent, unless dismissed by the family she is attending.

19—The nurse is required to keep the sick chamber neat, to see that everything for the patient's use is clean, and to perform any offices which the exigencies of the case and of particular occasions may demand, but she is not expected to do the work of a house servant.

20—The nurse is to remember that her office is not only to carry out the instructions of the medical attendant, but, what is equally important, to supplement his professional skill by patience, kindness of manner, and consideration for the sick; to be watchful, gentle, cleanly, and punctual, and, so far as prudence may allow, to study the wishes of those who are under her care.

21—The nurse is also enjoined to abstain altogether from repeating to her patient the opinions of the medical adviser as to the state of the invalid, serious impediments to recovery and unnecessary alarm having frequently followed such a departure from her specific duties.

22—The nurse is to bear in mind that inasmuch as her position is one of confidence, she is ever to hold sacred such family matters as may in the course of her attendance upon the sick come to her knowledge.

23—Ladies who desire to learn nursing, but do not wish to be bound for three years, may do so on their agreeing to comform to the rules, and paying for the first six months 10*s.* 6*d.* per week to the funds of the institute.

If after this period they are found suitable for nurses, they may either complete three years' training without further payment, providing their own uniform and paying for their washing; or they may be engaged on the usual terms.

24—The lady superintendent of nurses shall have the entire charge and management of the institute, and of the nurses, subject to the supervision of the institute committee; and so far as regards the nursing of the hospital, subject to the general control of the house-surgeon. She may, with the concurrence of the house-surgeon, suspend any nurse until a meeting of the institute committee, who n cases of urgency have the power of summary dismissal.

### Wages and Washing.

PART I.

Now we are going to the washing-tub again ; and we are going quite openly, and in spite of the sneers of the British nurse, whose squeamishness is even more laughable than that of the British matron, who annually amuses the public at the opening of the Royal Academy. The fact is, that washing is part of a nurse's wage, and the question of the payment a nurse receives for her labours is a very serious one, especially in the provinces. There are few outsiders who have the least idea of how serious a question this one of wages is, for the nurses who come prominently before the public are generally well-educated women with private incomes, who do not depend on their labours to provide them with daily bread. But out of 15,000 nurses there are only about one thousand who are thus luckily provided for, and in the interests of the other 14,000 we propose to give a few facts and statistics as to the money value of the skilled art of nursing.

NURSES V. SERVANTS' WAGES.—We all know how Tennyson's scorn is poured on the man who holds his wife "a little better than his dog, a little dearer than his horse;" and hospital authorities in many places seem to regard their nurses in much such a light; as "a little lower than the scrubber, and much below the servant-maid." The following table will show the relative value of nursing and domestic service in 14 hospitals :—

| Name of Hospital. | Nurses' Wages per annum. | * Upper Servants' Wages per annum. |
|---|---|---|
| PROVINCIAL. | | |
| Edinburgh Royal Infirmary | £20—30 | £14—40 |
| Leeds General Infirmary | 14—22 | 15—40 |
| Newcastle Infirmary | 20—27 | 20—30 |
| Nottingham General Hospital | 16—20 | 20—30 |
| Colchester Infirmary | 16—25 | 22—25 |
| Royal Hants County Hospital | 20 | 18—28 |
| Victoria Hospital, Burnley | 15 | 16—24 |
| Taunton and Somerset Hospital | 14—18 | 16—22 |
| Warneford Hospital | 15—25 | 14—32 |
| Chalmer's Hospital, Banff | 12—14 | 10—14 |
| LONDON HOSPITALS. | | |
| Middlesex | 18—24 | 26—35 |
| London Temperance | 20—25 | 18—30 |
| Heart Hospital, Soho Square | 15 | 16—30 |
| Chelsea Hospital for Women | 20—25 | 12—25 |

In the above table it will be seen that at Nottingham the lowest wage offered to a servant is the highest offered to a nurse.

PROBATIONERS V. SCRUBBERS' WAGES.—The following table will show the relative value put on the labours of scrubbers and probationers in 14 hospitals :—

| Name of Hospital. | Probationer's Wages. | Ward-maid's Wages. |
|---|---|---|
| PROVINCIAL. | | |
| Leeds General Infirmary | £10 | £12 |
| Victoria Hospital, Burnley | 9 | 12 |
| Warneford Hospital | 10 | 6—13 |
| Royal Berks Hospital | 10 | 10—16 |
| Devonshire Hospital, Buxton | 10—12 | 11—14 |
| Gravesend Hospital | 10 | 11 |
| Lancaster Infirmary | 13 | 14 |
| Warrington Infirmary | 12 | 12—14 |
| Nottingham General Hospital | 10 | 12 |
| Halifax Infirmary | 12—16 | 14 |
| Hull Royal Infirmary | 10 | 12 |
| Carmarthenshire Infirmary | 8 | 10 |
| Edinburgh Royal Infirmary | 10 | 10—16 |
| Leith Hospital | 10 | 12—14 |

In the greater number of hospitals the nurses and servants receive about the same wages; yet where a sister receives £30, you will often find the cook receives £40; and where a nurse receives £18, the servants generally receive £20; and where probationers have £10, the ward-maids will probably have £12. Of course there are numerous things which qualify these figures. Statistics are always more or less misleading : for instance, there is board, uniform, and washing. But we have not space to get to the washing-tub to day, so we shall have to continue our account of a nurse's worth and wages another week.

* Cooks and Housemaids, are included under "Upper Servants."

### Everybody's Opinion.

*Correspondence on all subjects is invited. No communications can be entertained if the name and address of the correspondent is not given, or unless one side of the paper only be written on.]*

### MILDMAY NURSES.

THE Superintendent (Mr. Bagster) writes : Your friendly notice in the last number of "The Nursing Mirror" of our Mildmay Deaconesses and Nurses is not quite accurate in its details as to the work done by the staff of the Nursing Home, and on one point might prove disadvantageous to our interests. Out of our staff of 104 nurses about 34 are engaged in the hospitals, &c., and the remaining 70 are constantly working in private families. The Mildmay Institutions are, I think, unique in this : That there are three small general hospitals—at Mildmay, Bethnal Green, and Jaffa—worked in connexion with the more ordinary operations called "mission work." At the last two of these hospitals there are a large number of out-patients, and in two other districts—Walworth and Victoria Park—there are also dispensaries, so that five physicians are wholly, and one partially, engaged in the work amongst the very poor.

### NURSES AND PRESENTS.

T. W. writes:—Most nurses who have read "Handclasp's" remark on "Presents to Nurses" must feel it is all they would wish to say ad they written on the subject, and I feel little can be added to it. I, for one, would only be too pleased could anything be done to do away with the system of giving nurses presents. I am not tied by any rule, but I always refuse if money is offered to me. In fact, I feel hurt, that my patients should think I require any payment besides my fee. I certainly have had books given me, mostly of a religious character—those one could not refuse for more reasons than one, but certainly unless there is a love for nursing, not any woman should take it up for the sake of a livelihood. I always feel fully repaid when I see my patient restored to health. I feel with "Handclasp," there are so many things more valued than presents, *i.e.*, consideration which a nurse does not always meet with. When any of my patients have talked of the way they have "made it up to nurses," I always express my disapproval of the system. Looking at the subject from the lowest standard, we are paid for our work and time, and it is only our duty to do all we can to alleviate the suffering of mind and body of our patients. Such a subject does not help to raise the profession which we follow, which is a most noble one, and calls for a great deal of self-sacrifice which only nurses know, and it seems a pity anything should be done to demoralize it.

A. E. C. writes:—I think it would be a very bad thing were private nurses allowed to take money from their patients. It would tend to lower the tone of nursing, and the matrons know only too well what a struggle it has been and still is to keep up a high tone amongst nurses and probationers. The gift of a book is so much useless lumber, as neither in the "home" or in her box has a nurse room for more than purely necessary things. Cannot each "Home" make its own rules? Could not all the money offered to nurses be given to the "Home" for the benefit of the nurses, and then divided each year to each nurse in proportion to the time she has served? But no nurse should partake of the fund who has not nursed two years in the same institution. There is a great fear that the nurses, as a body, should grow grasping, because so few of us have anything but what we work for—those who have money and position have no business as "hospital nurses"—and on that ground alone it would be bad for nurses to receive presents of money. May I say a word on "Convalescent Homes for Nurses"? I think, when a nurse wants a change or rest, she is better away from all "nurses and nursing." It is very difficult to know what to do with a poorly nurse when she has already had her holiday, so few can afford to go home twice in a year, and few ladies think of asking a nurse for a few days' change, all honour to those who do. So nurse goes off duty for a day or two, but never out of the smell and sound of cases. Very few hospitals have any fund for drives, concerts, &c.; and what can a matron do? She is powerless in most cases to give her nurses any change or amusement, and the provincial matrons have no powerful friends to help us, as our more favoured (and justly popular) sister at Ormond Street has.

A SPECIALITY.—M. E. MORLEY writes: All experienced nurses and doctors recognise that the study and nursing of sick children is a decided speciality. A children's nurse must be very "understanding" in her dealings with the little ones to be successful. Observation, patience, gentleness, tact, skill, and love are essential. The cry of a sick child varies in a most curious manner, and conveys much valuable information to the intelligent nurse. In brain disease the cry is sharp and sudden. In bronchial diseases the cry is hoarse and whispering. During teething the child will wail and make a curious crowing noise. With pain in the head there is generally contraction of the brows. The advent of a fit may be noticed by the peculiar twitching of the face and hands. The advent of croup—such a deadly enemy—may be often prevented, as the child, when crying, will croak. This cry, once heard, is never mistaken by a nurse. These signs are all valuable. No time is ever wasted that is bestowed on the children. They are so often misunderstood, not studied enough. Life is so real to them. We know the pain and weary waiting in bed will not last for ever, but they do not, and it is a nurse's privilege to comfort the little child. A nurse has many a weary hour of watching by the cots of her patients, but to a true and earnest woman there is help and courage in the very weariness, knowing that her ministry is the truest imitation of Him who went about doing good, healing the sick, and blessing the little children, bidding us to become like them in sweet simplicity and trusting love.

CORRECTING AN ERROR.—SISTER ROSA writes: Will you allow me to correct an error in your paper of the 17th inst. by stating that I was not a candidate for the matronship of Charing Cross Hospital?

NIGHT DUTY.—PERMANENT NIGHT NURSE writes: I wish you would call attention to the hard fate of a night nurse in a country infirmary, who often for years spends the greater part of day in bed. I do not know if others find it so, but I find that my health is always affected by night duty. In the London Hospital, where I trained, we had to take three months' day and three months' night work alternately, and then I got along very well; but now I have undertaken permanent night duty, and find it depressing and wearisome in the extreme. There is little chance of learning anything from the doctors, there is still less chance of amusement or seeing one's friends. Surely permanent night duty should be relegated to the superstitions of the past? I should be glad to know from any of your readers if they have ever undertaken and made prosper this eternal turning of night into day.

MENIAL WORK.—T. A. writes: In reply to your question whether we should consider it menial work for a nurse to drive an ambulance waggon, I would say, Most certainly not, when circumstances necessitated her doing it. It is really quite refreshing to hear of such a sensible woman as the Churchtown nurse in these times, when we are hearing so much nonsense about upholding the dignity of a nurse's position. Nothing should be deemed menial which a nurse does to procure the comfort and wellbeing of her patient. There is nothing more derogatory in scrubbing the floor, to keep his room sweet and clean, than there is in making a poultice to relieve his pain, and yet what awful snubbings a nurse often has to endure when it is known by her fellow-workers that she is always ready to make herself useful. In a small house, where she may have been sent to nurse, the same unpleasantness happens to the ward nurse, who will do what others consider menial in order to keep her ward in perfect cleanliness. However good a ward servant may be, if every item of work be left to her, it is impossible for everything to progress satisfactorily. Unfortunately, many excellent nurses spoil themselves by their persistent objection to do anything which they consider menial. In conclusion, I would say with you, that I consider the Churchtown nurse a woman worthy of everyone's sincere admiration.

### The Nurses' Bookshelf.

[Books for Review should be sent to The Editor, The Lodge, Porchester Square, W.]

### A Day with a District Nurse.*

THIS is a very brightly-written little pamphlet which ought to obtain its object of arousing interest in the Sick Poor Nursing Association of Manchester. The author started at nine one morning with one of the nurses, and looked on while the usual round of visits was made. She gives an exact and amusing description of all she saw, and then demands more help for the association that they may send nurses to those districts which yet lack their aid. The pamphlet concludes with a few sensible words we cannot do better than quote: "To me it seems the most perfect of philanthropic works, because it is not only helpful but most truly educational. It is a good thing to teach a man in hospital, that order, and decency, and cleanliness, are wholesome and pleasant things, and a hospital patient will often say, 'I wish I could have things like this at home.' But if you prove to him that he *can* have it at home, by simply using rightly the materials at hand, and making the best instead of the worst of things—if you *show* him his home made healthy and clean, without any alteration of circumstances, but simply by the exercise of some labour and judgment, you give a much more emphatic lesson."

* By A. L. B. A. Ireland and Co., Manchester. Price 1d.

### Appointments.

[It is requested that successful candidates will send a copy of their applications and testimonials, with date of election, to THE EDITOR, The Lodge, Porchester Square, W.]

ALDERSHOT LOCK HOSPITAL. — Miss E. E. Glanville is appointed Matron of the Aldershot Lock Hospital, and will reside there after November 27th. Miss Glanville was trained at the Royal Berks Hospital, Reading, and has been nursing for that institution for upwards of two years.

PORT ELIZABETH HOSPITAL, CAPE COLONY.—Nurses Annie Cameron and Alice Wood, of the Glasgow Royal Infirmary, sail in December to assume duty in the hospital at Port Elizabeth, and we wish them good speed. Seven nurses have now been sent by the matron of the "Royal," in response to applications from the Port Elizabeth Hospital authorities, and it must be gratifying to her to know that those who had previously gone out have given so much satisfaction as to induce others to be sent for.

### Vacancies.

**The following vacancies are announced:—**

### Lady Superintendent.

Nurses' Institute, Canterbury. Royal United Hospital, Bath; £60.

### Housekeeper.

Sheffield General Infirmary; £40.

### Nurses.

Nursing Institution, 96, 97, 98*a*, Wimpole Street, London, W., Mr. Wilson has several vacancies for thoroughly-trained Nurses.

Croydon General Hospital; two.

Establishment for Invalids, Harley Street; £25.

West Bromwich District Hospital, £22.

National Hospital for Paralysis, Queen Square; £20.

Home Hospital, Eastbourne.

Kingston Home, Hull.

Cheltenham Nursing Institution.

Tunbridge Wells Hospital; £22.

Nottingham and Notts Nursing Institute; £25; several.

Nursing Institute, Ryde; several.

Newport (Mon.) County Infirmary.

Kent Nursing Institution, West Malling; several.

Christ's Hospital, Hertford; £20.

Lewisham Union; £20; two.

Hospital for Paralysis, Portland Terrace; £20.

Guildford County Hospital; £12.

Stoke Newington Invalid Asylum, £25.

Royal Albert Hospital, Devonport.

Wrexham Union; £25.

Victoria Home, Bournemouth; £25.

Nurses' Home, Beckenham.

Nottingham Institution; £25.

Nurses' Institute, Canterbury.

Home for Epileptics, Maghull.

Brecon Infirmary; £18.

Mr. Wilson's Institution; several.

Royal Hospital for Children and Women, Waterloo Bridge Road; several.

Swansea and South Wales Nursing Institute; several.

Barnhill Hospital, Glasgow; £24.

Home Hospital, Fitzroy House, Fitzroy Square; several.

### Masseuse.

Cardiff Infirmary.

### Midwife.

Children's Hospital, Gloucester; £21.

### District Nurses.

Peterborough. Glasbury. Dublin.

### Probationers.

Bootle Borough Hospital.

Workhouse Infirmary Nursing Association.

Kent Nursing Institution, West Malling; several.

Blackwall and Charlton Cottage Hospital.

Blackheath Cottage Hospital.

National Hospital for Paralysis, Queen Square.

Royal National Hospital for Consumption, Ventnor, I.W.; £10; several.

### Notes and Queries.

TO CORRESPONDENTS.—1. Questions may be written on post-cards. 2. Advertisements in disguise are inadmissible. 3. In answering a query please quote the number. 4. If a private answer is desired, a stamped addressed envelope must be forwarded.

### Answers.

(79) *Lectures on Nursing.*—You cannot do better than get Lückes Lectures on Nursing, from Churchill, price 2*s.* 6*d.* I have found them of the greatest help.—*T.A.*

(85) *Doris* could teach her patient to make comforters, &c., on a frame. Both writing and drawing can be done with one hand if the paper is fastened to a board with four pins.

*Rotunda Hospital.*—Pupils in Midwifery are only taken for six months at least. Fee £10 for teaching, or £20 for residents in hospital.—*G. Seymour.*

